# Cell type specific gene expression profiling reveals a role for complement component C3 in neutrophil responses to tissue damage

**DOI:** 10.1038/s41598-020-72750-9

**Published:** 2020-09-24

**Authors:** Ruth A. Houseright, Emily E. Rosowski, Pui-Ying Lam, Sebastien J. M. Tauzin, Oscar Mulvaney, Colin N. Dewey, Anna Huttenlocher

**Affiliations:** 1 Department of Pathobiological Sciences, University of Wisconsin-Madison, Madison, WI USA; 2 Department of Biological Sciences, Clemson University, Clemson, SC USA; 3 Department of Pharmacology and Toxicology, University of Utah, Orem, UT USA; 4 Department of Biology, Utah Valley University, Orem, UT USA; 5 Department of Genetics, University of Wisconsin-Madison, Madison, WI USA; 6 Department of Biostatistics and Medical Informatics, University of Wisconsin-Madison, Madison, WI USA; 7 Department of Medical Microbiology and Immunology, University of Wisconsin-Madison, Madison, WI USA; 8 Department of Pediatrics, University of Wisconsin-Madison, Madison, WI USA

**Keywords:** Cell migration, Cell biology, Immunology

## Abstract

Tissue damage induces rapid recruitment of leukocytes and changes in the transcriptional landscape that influence wound healing. However, the cell-type specific transcriptional changes that influence leukocyte function and tissue repair have not been well characterized. Here, we employed translating ribosome affinity purification (TRAP) and RNA sequencing, TRAP-seq, in larval zebrafish to identify genes differentially expressed in neutrophils, macrophages, and epithelial cells in response to wounding. We identified the complement pathway and *c3a.1*, homologous to the C3 component of human complement, as significantly increased in neutrophils in response to wounds. *c3a.1*^−/−^ zebrafish larvae have impaired neutrophil directed migration to tail wounds with an initial lag in recruitment early after wounding. Moreover, *c3a.1*^−/−^ zebrafish larvae have impaired recruitment to localized bacterial infections and reduced survival that is, at least in part, neutrophil mediated. Together, our findings support the power of TRAP-seq to identify cell type specific changes in gene expression that influence neutrophil behavior in response to tissue damage.

## Introduction

Acute tissue injury is characterized by a rapid influx of both neutrophils and macrophages into the wound microenvironment, followed by inflammatory resolution and wound healing. This initial recruitment of leukocytes to the wound is of critical importance; neutrophils, the most abundant cell type and the first responders to tissue damage, limit infection at the wound site^1^, while macrophages remove debris that would otherwise impede the repair process^2^. In addition, neutrophils in the wound microenvironment play complex roles in wound healing. They contribute directly to wound healing by producing mediators that promote angiogenesis and recruitment of keratinocytes and fibroblasts that are needed for repair^3^, and the specific importance of neutrophils to efficient wound repair is illustrated by delays in skin wound healing reported in neutropenic patients^4^. However, neutrophils can also delay repair and contribute to tissue injury by amplifying the local inflammatory response and by releasing toxic mediators and neutrophil extracellular traps^5^.

In order for efficient recruitment to the wound to occur, leukocytes must sense and respond to a complex milieu of signals, both from the damaged tissue itself and from one another. Previous studies have shown that neutrophils respond to recruitment signals such as G-coupled protein receptor (GPCR) engagement and exposure to bacterial formyl peptides and lipopolysaccharides with rapid changes in gene transcription^6,7^. Similarly, functional specialization of mature macrophages by activation of pro-inflammatory or pro-resolving transcriptional programs is a well-established phenomenon^8^. However, the cell type-specific transcriptional changes that occur during innate immune cell recruitment and wound healing and their effects on leukocyte migration have not yet been comprehensively characterized.

We have previously reported the use of translating ribosome affinity purification (TRAP) to detect changes in gene expression in specific cell types resulting from heat shock in zebrafish^9^. However, this method has not, to our knowledge, been used to detect cell type-specific differential gene expression in response to wounding. Using this method, we identified several hundred differentially expressed genes, including upregulation of complement system components.

As a non-cellular arm of the innate immune system, the complement system is classically considered to be an extracellular pathogen sensor of the innate immune system and plays a critical role in mediating leukocyte function. Critical complement proteins such as C3 and C5 are produced by the liver and circulate in serum^10,11^. Activation of complement via either the classical, alternative, or mannose-binding lectin pathways results in a cascade of proteolytic cleavages of complement components, converging at the hydrolysis and activation of component C3 to C3A and C3B. C3B subsequently binds to other complement pathway proteins to form C5 convertase, which cleaves component C5 to C5A and C5B^10,11^. Cleavage of C3 and C5 permit the effector functions of complement, including opsonization and phagocytosis of microorganisms, direct microbial killing by the formation of the membrane attack complex, and enhanced cellular immune responses^10^.

While serum-effective C3 and C5 play canonical roles in antimicrobial defense, and extracellular C5A is known to be a powerful chemoattractant for neutrophils^10^, the effect of innate immune cell-derived complement components remains unclear. Work within the last decade has demonstrated the transcription of intracellular complement components within multiple cell types, including T cells, monocytes and macrophages, and epithelial cells^12^. This intracellular “complosome” functions independently of serum-associated complement proteins and plays key noncanonical, autocrine roles in both host defense and basic cellular functions^12,13^. For example, in CD4+ T cells, transcription of C3 is triggered by integrin signaling during diapedesis and licenses these cells for Th1-type effector functions^14^. Although neutrophils contain intracellular C3 and macrophages transcribe C3 in response to inflammation^15^, a specific role for intracellular C3 in the recruitment and migration of innate immune cells has not yet been described.

Zebrafish represent a strong in vivo system to answer these questions, as they have functioning cellular and noncellular arms of the innate immune system that are largely conserved to those of humans, including neutrophils^16^, macrophages^17,18^, and the complement system^19^. The zebrafish complement system is structurally and functionally similar to that of humans, and zebrafish express homologs to all of the fundamental mammalian complement components^19,20^. Zebrafish have high fecundity, which increases the statistical power of experiments, and are thus an attractive model for use in large-scale genetic and drug screens. Finally, zebrafish are highly amenable to live imaging of leukocyte migration in response to inflammatory stimuli^21^.

In this work, we report that cell-specific TRAP-RNAseq of larval zebrafish identifies genes differentially expressed in neutrophils, macrophages, and epithelial cells in response to wounds. Our data identify upregulation of the complement pathway in all cell types, with specific, statistically significant upregulation of *c3a.1*, homologous to the C3 component of human complement, in neutrophils. We find that mutation of *c3a.1* in larval zebrafish impairs neutrophil recruitment to localized bacterial infection and show a neutrophil-dependent role for C3 in survival in response to these infections. We further show that mutation of *c3a.1* impairs neutrophil migration to wounds and impairs wound healing. Finally, we show that the defect in neutrophil recruitment is likely due to decreased neutrophil migratory speed in the early post-wounding period. Together, our findings suggest a role for C3 in optimizing neutrophil responses to tissue injury by priming neutrophils to respond to other inflammatory cues.

## Results

### TRAP-RNAseq identifies genes differentially regulated in neutrophils, macrophages, and epithelial cells in response to wounding

**Figure 1:**
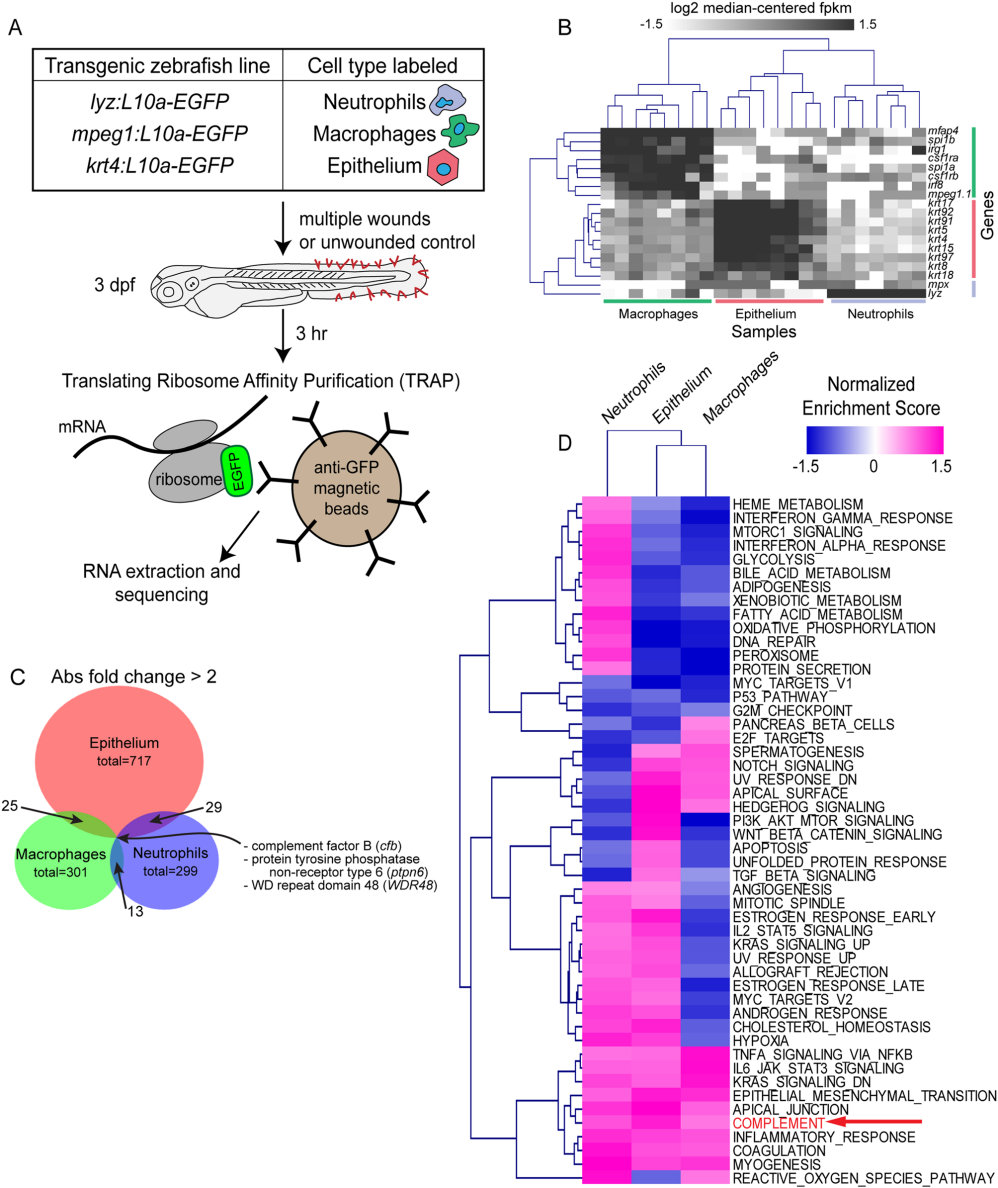
TRAP-RNAseq identifies differential expression of genes by neutrophils, macrophages, and epithelial cells in response to wounding. (**A**) Experimental setup. 3 dpf transgenic zebrafish larvae expressing an EGFP-tagged copy of the ribosomal subunit L10a specifically in neutrophils (*lyz*), macrophages (*mpeg1*), or epithelium (*krt4*) were subjected to multiple fin tissue wounds. 3 h later, larval tissue was homogenized and ribosomes were isolated with α-GFP immunoprecipitation. RNA was then extracted and subject to Illumina sequencing. (**B**) Expression of a priori tissue-specific genes across all analyzed samples. Columns represent samples, labeled by cell type-specific promoter used; rows represent known cell-type-specific genes. (**C**) Venn diagram of genes found to be more than twofold changed by wounding in each cell type. (**D**) Normalized enrichment scores of Molecular Signatures Database Hallmark Gene Sets (rows) in each cell type (columns), from Gene Set Enrichment Analyis (GSEA).

Multiple cell types, including neutrophils, macrophages, and epithelial cells, respond to tissue injury with rapid transcriptional changes that aid in host defense and wound healing. However, the relative transcriptional contributions of each cell type are incompletely understood. To identify cell-specific signals that are differentially expressed in different cell types in response to wounding, we performed a large-scale translating protein affinity purification and RNA sequencing (TRAP-RNAseq) screen (Fig. 1A). Briefly, 3 dpf transgenic zebrafish larvae expressing an EGFP-tagged copy of the ribosomal subunit L10a specifically in neutrophils, macrophages, or epithelium were subjected to multiple fin tissue wounds. 3 h later, larval tissue was homogenized and ribosomes were isolated with α-GFP immunoprecipitation. RNA was then extracted. Illumina sequencing confirmed expression of a priori-selected, known cell-type-specific genes across all analyzed samples, validating our method (Fig. 1B). We then focused our analysis solely on zebrafish genes that have identified human homologs. From these genes, 299 were identified to be at least twofold changed (upregulated or downregulated) in neutrophils, 301 in macrophages, and 717 in epithelial cells (Fig. 1C). It is interesting to note that relatively few genes were more than twofold differentially expressed in more than one cell type (Fig. 1C and Supplemental Table 1), suggesting that wounding-specific transcriptional programs differ across cell types, even among acute inflammatory cells. Gene set enrichment analysis (GSEA) of Hallmark genes from the Molecular Signatures Database to identify groups of genes sharing a common biologic function^22^ further showed enrichment of genes involved in the complement pathway in wounded fish, compared with unwounded controls (Fig. 1D).

### TRAP-RNAseq identifies *c3a.1* and other complement components as factors upregulated upon wounding

**Figure 2:**
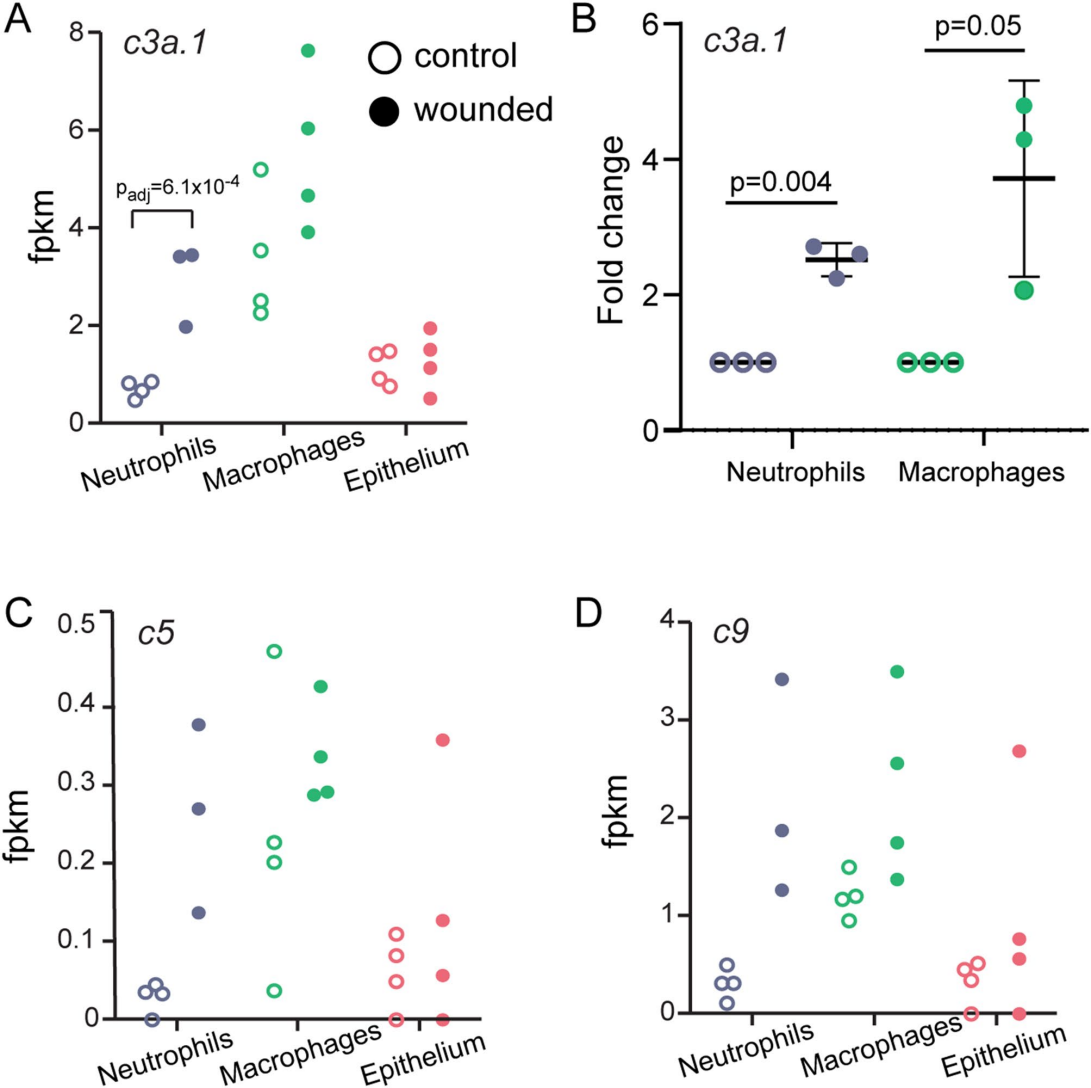
TRAP-RNAseq identifies upregulation of the complement pathway and *c3a.1* in response to wounding. (**A**) Expression measured by RNA-seq (fpkm) of complement pathway gene *c3a.1* across all three cell types. Each dot represents one replicate. (**B**) RT-qPCR validation of *c3a.1* gene expression in neutrophils and macrophages. Each dot represents one replicate; measurements for each replicate were performed in triplicate. Data is expressed as the fold change in mean Cq, normalized to *ef1a* expression and expression of *c3a.1* in unwounded controls. N = 200 larvae/condition/replicate. C, D. Expression measured by RNA-seq (fpkm) of complement pathway genes *c5* (C) and *c9* (D), across all 3 three cell types. Each dot represents one replicate.

Among the differentially expressed genes we identified, *c3a.1* showed a statistically significant increase in mRNA expression in neutrophils following wounding (Fig. 2A). A non-significant increase in *c3a.1* was also evident in macrophages. We confirmed these findings by TRAP-RT-qPCR and detected a statistically significant 2.24 to 2.71-fold increase in *c3a.1* expression by neutrophils at 3 hpw (Fig. 2B). A non-significant 2.07 to 4.79-fold increase was present in macrophages at 3 hpw. Other complement pathway components, including *c5* and *c9*, also showed trends toward increased expression in neutrophils and macrophages (Fig. 2C,D). Further, complement factor B (*cfb*) was one of only 3 genes that were differentially expressed in all 3 cell types (Fig. 1C). Taken together, these data suggest an important role for the complement pathway in general, and *c3a.1* in particular, in orchestrating innate immune responses in the context of wounding.

### Validation of *c3a.1*-deficient zebrafish lines

**Figure 3:**
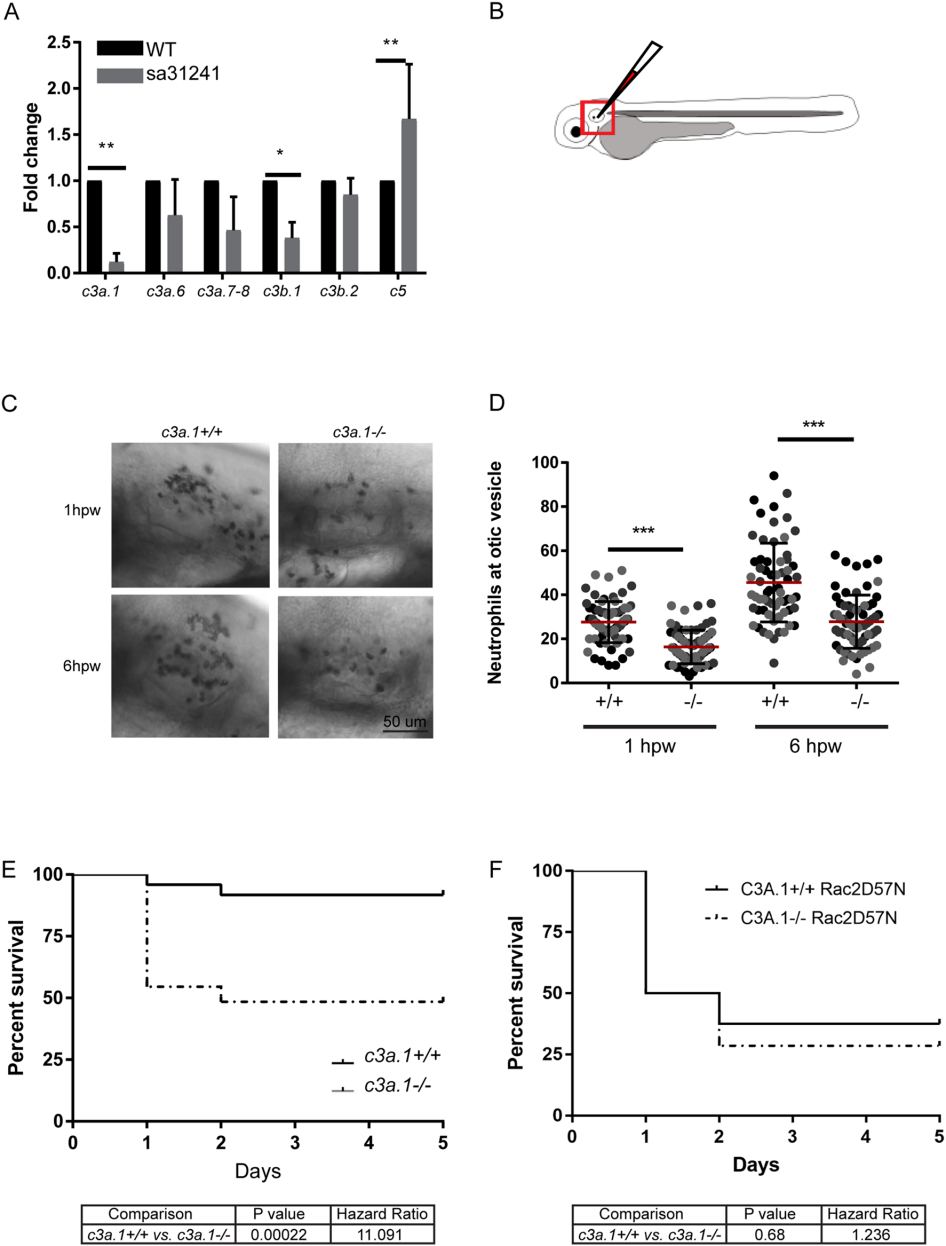
Global depletion of *c3a.1* decreases neutrophil recruitment to, and subsequent survival of, localized bacterial infection. (**A**) RT-qPCR validation of *c3a*, *c3b*, and *c5a* orthologue expression in pooled WT and *c3a.1*^*−/−*^ (sa31241) whole zebrafish larvae, normalized to WT expression for each gene and to *ef1α*, with data expressed as mean + /- SEM. Data comprise 3 independent experiments, performed in triplicate, n = 50 larvae per condition per experiment. (**B**) Experimental setup. *c3a.1*^+/+^ (n = 71, 1 hpw; 65, 6 hpw) and *c3a.1*^−/−^ (n = 71, 1 hpw; 68, 6 hpw) larvae were inoculated with 1000 CFU *Pseudomonas aeruginosa* in the left otic vesicle and subsequently stained with Sudan Black B. The otic vesicle region (box) was imaged. (**C**) Representative images and (**D**) quantification of neutrophil recruitment following otic vesicle infection, with data expressed as mean + /- SEM. Each dot represents one larva; colors represent results of 3 independent experiments. ***p < 0.001 (E) *c3a.1*^+/+^ (n = 24) and *c3a.1*^−/−^ (n = 32) larvae were infected with 7500 CFU *Pseudomonas aeruginosa* in the left otic vesicle and survival was tracked over 5 days post-infection. (F) To test whether survival was neutrophil-dependent, *c3a.1*^+/+^ (n = 16) and *c3a.1*^−/−^ (n = 14) larvae with neutrophils that are mcherry-labeled and carry a mutation in *rac2* rendering them migration-defective (*Tg*(*mpx:rac2*^*D57N*^*-mcherry*)) were infected with *Pseudomonas aeruginosa* as in C and survival was tracked over 5 days post-infection. E and F each comprise 3 independent experiments.

C3a.1 shares an approximately 43% amino acid similarity to the human C3 component of complement^23^. In order to investigate the role of *c3a.1* in leukocyte responses in the context of wounding, we obtained zebrafish expressing an A to T nonsense mutation in exon 22 of 41 of the *c3a.1* sequence, producing a premature stop codon (sa31241, Sanger)^24^. This premature stop codon occurs prior to the predicted thioester bond and α2 macroglobulin-complement domains of the C3A protein that characterize an anaphylatoxin^25^. qPCR of cDNA from pooled 3 dpf *c3a.1*^*−/−*^ larvae confirmed loss of *c3a.1* mRNA, compared with *c3a.1*^+*/*+^ controls, and showed no significant compensatory upregulation of the other *c3a* orthologues expressed at this stage of larval development (Fig. 3A). Amplification of *c3a.2-3, c3a.4,* and *c3a.5* by RT-qPCR was not observed; this is in agreement with existing reports that these orthologues are not expressed in unwounded 3 dpf larvae^23^. Expression of other major complement pathway genes based on qPCR of cDNA from pooled 3 dpf *c3a.1*^*−/−*^ larvae was similar to that of *c3a.1*^+*/*+^ controls: although a modest, non-significant decrease in *c3b.1* mRNA was noted, *c3b.2* mRNA was unchanged and a significant, potentially compensatory, increase in *c5* mRNA was observed (Fig. 3A).

### Global depletion of *c3a.1* decreases neutrophil recruitment to, and survival of, localized bacterial infection

The complement system plays a canonical role in pathogen recognition and clearance, and patients with deficiencies in the classical complement pathway are susceptible to pyogenic infections^26^. Thus, we first measured the ability of neutrophils in *c3a.1-*deficient larvae to migrate to localized bacterial infections. Using an established model of localized *Pseudomonas aeruginosa* infection of the otic vesicle (Fig. 3B)^27^, we found that *c3a.1*^*−/−*^ larvae had fewer neutrophils at the site of the infection at both 1 h post-infection (hpi) and 6 hpi, compared with *c3a.1*^+*/*+^ controls (Fig. 3C,D). Neutrophils are believed to be the main cell type responsible for resistance to *Pseudomonas* infections^28^. Consistent with the defect in neutrophil recruitment we observed, we found that *c3a.1*^*−/−*^ larvae had increased susceptibility to *Pseudomonas* infection, with ~ 50% of infected larvae dying by only 1 dpi. In contrast, > 95% of *c3a.1*^+*/*+^ larvae survived to 5 dpi (hazard ratio: *c3a.1*^*−/−*^ vs. *c3a.1*^+*/*+^  = 11.091) (Fig. 3E).

### *C3a.1* mediates resistance to *Pseudomonas aeruginosa* infection in a neutrophil-dependent manner

Increased susceptibility to localized *Pseudomonas* infection in *c3a.1*^*−/−*^ larvae could be due to impaired neutrophil recruitment or function, and/or the loss of other complement-mediated effects, such as bacterial opsonization and direct lysis. To determine whether increased susceptibility to *Pseudomonas* infection in *c3a.1*^*−/−*^ larvae is due to neutrophil-intrinsic activity, we crossed the *c3a.1*-deficient line to the *Tg(mpx:mcherry-2A-rac2*^*D57N*^*)* line, in which mcherry-labeled neutrophils express a dominant negative form of Rac2 and are thus rendered migration-deficient. As we have previously reported^29^, *c3a.1*^+*/*+^ larvae with neutrophils expressing *rac2*^*D57N*^ have increased susceptibility to *Pseudomonas* infection, with ~ 50% mortality at 1 dpi. In comparison, we noted no significant change in susceptibility in *c3a.1*^*−/−*^ larvae with neutrophils expressing *rac2*^*D57N*^, compared with *c3a.1-* intact *rac2*^*D57N*^ larvae (HR *c3a.1*^*−/−*^* rac2*^*D57N*^ vs. *c3a.1*^+*/*+^ *rac2*^*D57N*^ = 1.236) (Fig. 3F). Therefore, the increase in susceptibility to *Pseudomonas* infection we observed in *c3a.1*^*−/−*^ larvae expressing wild-type *rac2* is likely predominantly due to a neutrophil-intrinsic function of C3, possibly due to the reduction in numbers of neutrophils found at the infection site.

### Global depletion of *c3a.1* decreases neutrophil recruitment to wounds

**Figure 4:**
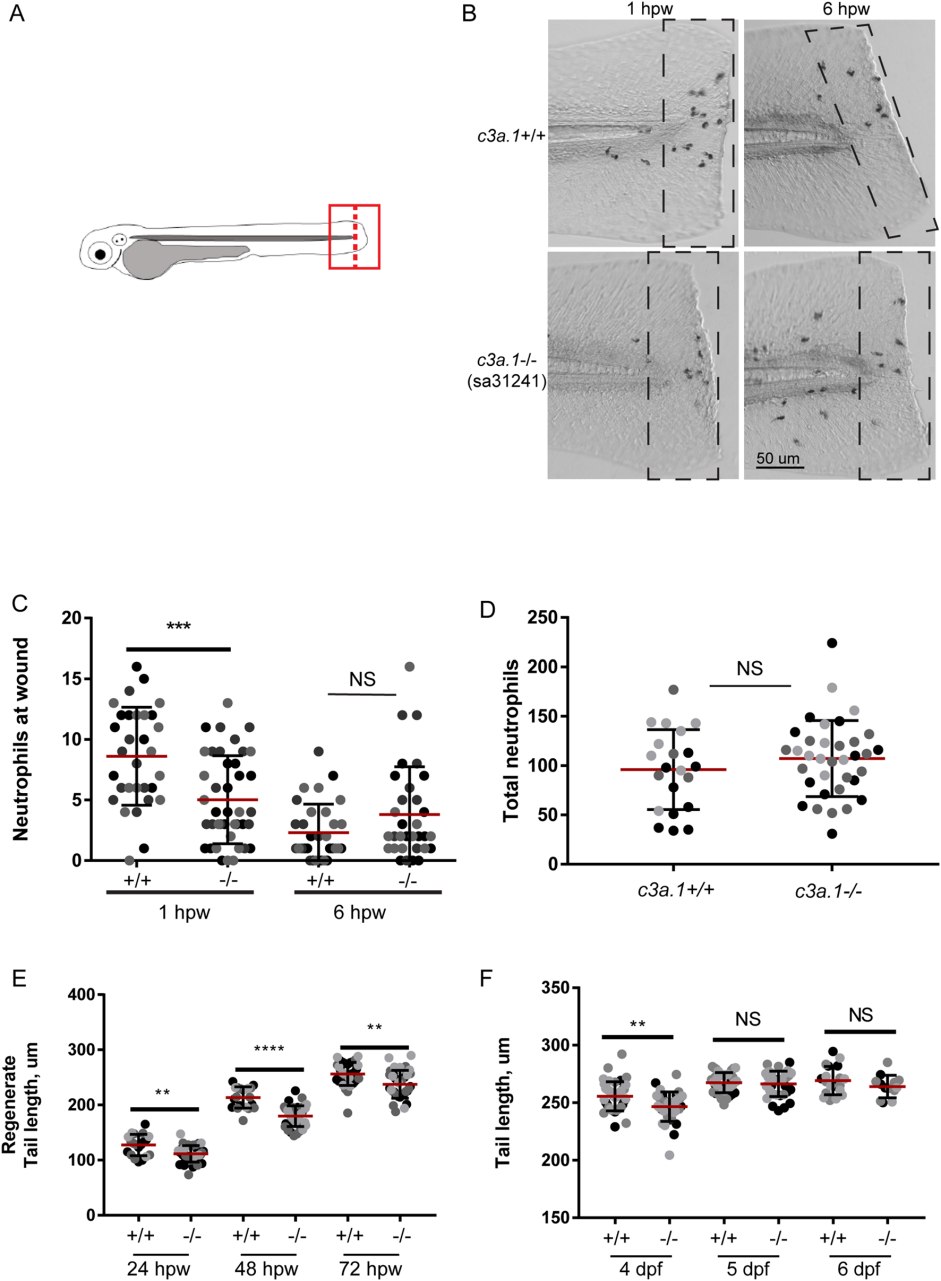
Global depletion of *c3a.1* decreases early neutrophil recruitment to a wound. (**A**) Experimental setup. 3 dpf *c3a.1*^+*/*+^ or *c3a.1*^*−/−*^ zebrafish larvae were subjected to wounding by tail transection (dashed line), subsequently stained with Sudan Black B, and the tail region (box) was imaged. (**B**) Representative images and (**C**) quantification of neutrophil recruitment following tail transection in *c3a.1*^+*/*+^ (n = 31, 2 hpw; 32, 8 hpw) and *c3a.1-/-* (n = 41, 2 hpw; 33, 8 hpw) larvae. (**D**) Quantification of total neutrophil counts in *c3a.1*^+*/*+^ (n = 21) and *c3a.1*^*−/−*^ (n = 35) larvae. (**E**) Quantification of caudal fin regenerate length following tail transection, 24 hpw-72 hpw, of *c3a.1*^+*/*+^ (n = 20, 24 hpw; 20, 48 hpw; 36, 72 hpw) and *c3a.1*^*−/−*^ (n = 39, 24 hpw; 40, 48 hpw; 36, 72 hpw) larvae. (**F**) Quantification of caudal fin length during larval development, 4dpf-6dpf, of *c3a.1*^+*/*+^ (n = 40, 4dpf; 40, 5dpf; 19, 6dpf) and *c3a.1*^*−/−*^ (n = 36, 4dpf; 34, 5dpf; 13, 6dpf) larvae. For **C**–**F**, all data are expressed as mean + /− SEM; each dot represents one larva; colors represent results of 3 independent experiments. *p < 0.05, **p < 0.01, ***p < 0.001, ****p < 0.0001.

Because *c3a.1* expression is significantly increased in neutrophils in response to wounding, we next investigated the neutrophil response to wounding in *c3a.1*^*−/−*^ larvae. Compared with *c3a.1*^+*/*+^ controls, *c3a.1*^*−/−*^ larvae had significantly decreased numbers of neutrophils in the wound microenvironment at 2 hpw (Fig. 4A–C). However, by 8 hpw, neutrophil numbers at the wound did not differ significantly between *c3a.1*^*−/−*^ and *c3a.1*^+*/*+^ larvae (Fig. 4B,C), suggesting that the neutrophil recruitment phenotype induced by *c3a.1* depletion is confined to the early post-wounding period. Changes in neutrophil numbers at the wound are due to a specific change in the recruitment response, as whole-larvae total neutrophil numbers did not differ between *c3a.1*^*−/−*^ larvae and *c3a.1*^+*/*+^ fish (Fig. 4D). These findings are in line with a neutrophil recruitment phenotype reported by Forn-Cuni, et al., in response to *c3a.1* knockdown using antisense morpholino^23^. Macrophage recruitment to tail transection wounds did not differ between *c3a.1*^*−/−*^ and *c3a.1*^+*/*+^ larvae (Fig. S1). In addition, *c3a.1*^*−/−*^ larvae displayed significantly decreased regenerate fin length at 24, 48, and 72 h post-wounding (equivalent to 4, 5, and 6 dpf, respectively), compared with *c3a.1*^+*/*+^ clutchmates (Fig. 4E), above and beyond the mild developmental foreshortening of the tail we noted in *c3a.1*^*−/−*^ larvae at 4dpf only (Fig. 4F). Taken together, these findings suggest that C3 modulates neutrophil wound responses and wound healing in zebrafish larvae.

### Depletion of *c3a.1* does not alter neutrophil egress from hematopoietic tissues following wounding

We next asked whether C3 controls neutrophil mobilization from hematopoietic tissue. In response to inflammatory signals, zebrafish neutrophils may be released directly from hematopoietic tissues or recruited from a population of neutrophils already patrolling in peripheral tissues^30^. C3A has been shown in mice to help to retain immature neutrophils in hematopoietic tissues^31,32^. C3 and C3A receptor-deficient mice subsequently have faster and more pronounced neutrophil egress from bone marrow in response to inflammatory stimuli^33^. Although this finding is opposite to the decreased neutrophil numbers that we observe at a wound in larval zebrafish (Fig. 4B,C), we still wanted to determine whether decreased neutrophil numbers at inflammatory sites in *c3a.1*^*−/−*^ larvae were due to a difference in neutrophil recruitment from hematopoietic tissue. At 3 dpf, the primary organ of hematopoiesis in the larval zebrafish is the caudal hematopoietic tissue (CHT), in which hematopoiesis resembles that within the mammalian fetal liver^34^. We crossed the *c3a.1*-deficient line to the *Tg(mpx:dendra2*) line, in which neutrophils are labeled with the photoconvertible fluorophore Dendra2, enabling fate tracking of neutrophils originating from the CHT over time ^35^. We photoconverted neutrophils in the CHT and then subjected the larvae to tail transection. We subsequently counted both the photoconverted neutrophils remaining in the CHT and those mobilized to the periphery at 3 hpw (Fig. S2A). Neither the number of neutrophils retained in the CHT nor the number mobilized neutrophils differed between *c3a.1*^+*/*+^ and *c3a.1*^*−/−*^ larvae (Fig. S2B,C). This suggests that decreased neutrophil numbers at the wound in *c3a.1*^*−/−*^ larvae are not due to alterations in neutrophil egress from the hematopoietic tissue and led us to more closely examine neutrophil interstitial migration.

### Loss of *c3a.1* impairs neutrophil migration speed early after wounding

**Figure 5:**
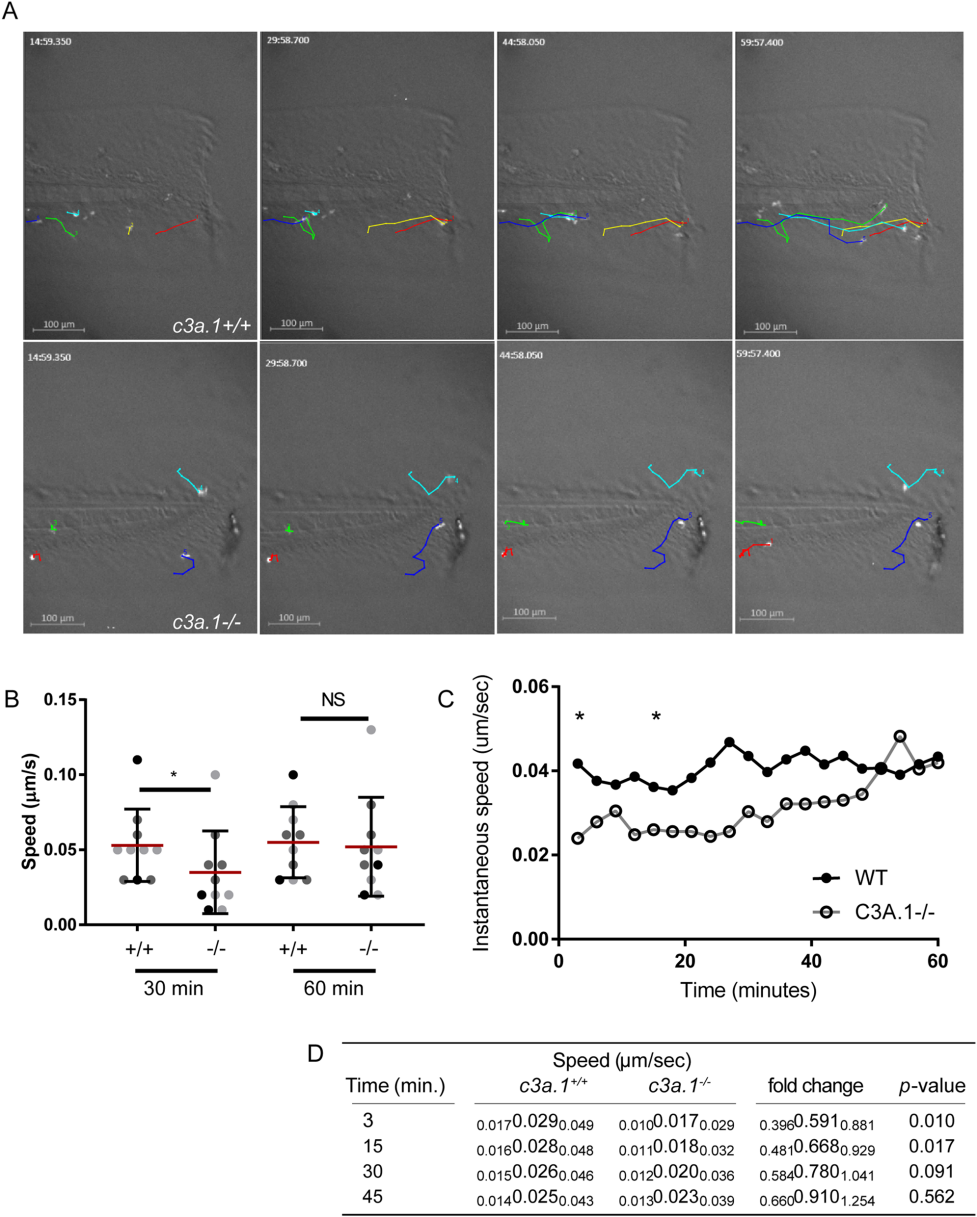
Loss of *c3a.1* impairs neutrophil recruitment by decreasing neutrophil migration speed early after wounding. (**A**) Time-lapse photomicrographs of neutrophil recruitment to tail-transected caudal fins of *c3a.1*^+*/*+^ (n = 8) or *c3a.1*^*−/−*^ larvae (n = 8) with mcherry-labeled neutrophils (*Tg(mpx:mcherry))*, 0–60 min post-wound, showing tracks of forward migrating neutrophils. Images with track overlay were generated using the MTrackJ plugin^68^ in ImageJ^69^ (**B**) Quantification of mean track speed of forward-migrating neutrophils, expressed as mean + /- SEM. Each dot represents the mean of the first 5 neutrophils recruited to the wound of an individual larva. Colors represent the results of 4 independent experiments. *p < 0.05 (**C**) Graph, expressed as mean, and (**D**) quantification of instantaneous speed of all forward-migrating neutrophils over the first 60 min following wounding. In (D), for speed and fold change, data are expressed as median (center values), with 95% confidence intervals (small print). Data comprise 4 independent experiments. *p < 0.05.

Our data thus far suggest a role for C3 in priming neutrophils to migrate effectively toward other chemotactic signals. Thus, we next tested whether the impaired neutrophil recruitment phenotype we observed in *c3a.1*^*−/−*^ zebrafish larvae was due to alterations in the dynamics of interstitial migration to the wound. We took advantage of the amenability of larval zebrafish to live time-lapse imaging and single-cell tracking to determine how the interstitial migration characteristics of neutrophils in *c3a.1*-deficient larvae differ from those of wild-type controls. To do this, we crossed the *c3a.1*-deficient line to the *Tg(mpx:mcherry)* line^36^, in which neutrophils express the fluorescent protein mcherry. Following tail transection, we imaged labeled neutrophils in the wound microenvironment for 1 h and tracked the neutrophils using Imaris software (Bitplane) (Fig. 5A and movie 1). We found that average neutrophil speed during the first 30 min after wounding is significantly impaired in *c3a.1*^*−/−*^ larvae, compared to *c3a.1*^+*/*+^ controls. This change in neutrophil migratory behavior is confined to the early post-wounding period, as when speed is averaged over the first 60 min post-wound, it is not different between groups (Fig. 5B). The mean displacement and track straightness traveled by the neutrophils also did not differ between groups (Fig. S3A-B). Decreased neutrophil speed in *c3a.1*^*−/−*^ larvae is specific to neutrophil directed migration, as neutrophil random migration in the absence of an inflammatory stimulus is not impaired in *c3a.1*^*−/−*^ larvae, and neutrophil random migration speed is in fact slightly faster in *c3a.1-*deficient zebrafish compared to controls (Fig. S3C). Finally, quantification of each neutrophil’s instantaneous speed at 3 min intervals over the first hour post-wounding shows that neutrophils in *c3a.1*-intact larvae rapidly achieve and maintain a steady speed toward the wound. In contrast, neutrophils in *c3a.1*^*−/−*^ initially migrate significantly more slowly, before accelerating to reach *c3a.1*^+*/*+^ speeds by about 30 min post-wound (Fig. 5C,D). Altogether, these data support the idea that C3 primes early neutrophil responses to damaged tissues.

## Discussion

Here we report, for the first time, the results of a cell type-specific translation profiling screen designed to identify genes differentially transcribed in the inflammatory context of wounding in the larval zebrafish model. We were surprised to find relatively little overlap in the transcriptomes of neutrophils, macrophages, and epithelial cells in that few genes identified by our screen were differentially expressed in more than one cell type. This suggests the presence of a complex network of inter- and intracellular signals, in which cross-communication among cell types is essential for optimal leukocyte recruitment after wounding.

We have previously shown that the signals that guide neutrophils to sites of sterile injury differ from those that regulate migration to bacterial infection; specifically that, while PI3K signaling is required in both contexts, tissue-generated H_2_O_2_ signaling is required for neutrophil responses to wounds, but is dispensable for neutrophil responses to infection^37^. Although there is increasing recognition in the field that molecular drivers of innate immune system responses are not universal, our work here supports the power of large-scale gene expression profiling to identify important regulatory pathways that are conserved across multiple types of inflammatory stimuli. Further screening and identification of context-dependent alterations in the transcriptomes of leukocytes and epithelial cells have the potential to uncover more genes that are differentially expressed only in response to a specific type of inflammatory stimulus. In either case, this work demonstrates the utility of large-scale translation profiling screens in zebrafish to identify promising genes for further study.

We identified *c3a.1* as significantly upregulated in neutrophils in response to wounding. This finding is interesting because, while the complement system has been implicated in multiple inflammatory contexts, including wounding, infection, and hematopoiesis, it is best understood in infection, where it functions to opsonize bacteria for phagocytosis or kill them directly via assembly of the membrane attack complex^10^. Similar to our finding that *c3a.1*^*−/−*^ zebrafish have impaired survival to bacterial infection, mice deficient in either C3 or the C3A receptor have increased susceptibility to septic arthritis^38^, and mice with C3A over-activation induced by deletion of the scavenger carboxypeptidase B2 displayed a survival benefit in the context of polymicrobial sepsis^39^, confirming a specific, protective role for C3A in infectious inflammatory contexts. However, we find that, in larval zebrafish, C3 acts through neutrophils, as C3 mutation had no further effect when neutrophils were defective.

The role of C3 in the context of sterile injury is less well understood. The complement pathway is a major driver in pattern formation during amphibian limb regeneration^40^. Furthermore, C3A signaling through the C3A receptor (C3AR) is required for hepatocyte proliferation and liver regeneration following toxic liver injury in mice^41^, and C3A can be detected in the wound microenvironment of incised skin wounds in guinea pigs^42^. Rafail et al. showed in 2015 that C3^*−/−*^ mice have faster early wound healing and decreased neutrophil recruitment at wounds than their wild-type counterparts^43^. However, while this work showed evidence of a role for the complement pathway in wound-associated inflammation and wound healing, the findings were attributed to a lack of downstream C5a-C5aR1 signaling rather than specific loss of C3 activation^43^. While a decrease in C5 activation in the *c3a.1*^*−/−*^ zebrafish we used in our study cannot be entirely excluded, zebrafish larvae express multiple homologs of C3^19^, and it seems unlikely that mutation of *c3a.1* alone abrogates C5 convertase activity in these fish. As neither full-length C3 or its cleaved anaphylatoxin C3A have been shown to act as neutrophil chemoattractants^44,45^, our findings suggest a novel, specific role for C3 in recruiting neutrophils to wounds. We further show that impaired neutrophil recruitment in *c3a.1*^*−/−*^ larvae is accompanied by impaired wound healing, evidenced by a decrease in tail fin regenerate length for up to 72 h post-tail transection and consistent with previous reports^23^ using morpholinos to knock down *c3a.1* expression. Previous research in our lab has shown that inhibition of transient signals such as hydrogen peroxide that stimulate neutrophil recruitment within the first 1–2 h after wounding can impair tail fin regeneration for up to 3 days^46^. Although it is likely that *c3a.1* has other direct or indirect effects on wound healing, our data suggest a potential role for C3 in linking neutrophil recruitment to tissue regeneration.

Our data raise additional questions about a neutrophil-specific, intracellular role for C3. Our data using *rac2*^*D57N*^ zebrafish mutants suggest that, during infection, impaired survival in *c3a.1*^*−/−*^ larvae is a neutrophil-dependent phenotype. Although we detected multiple C3 homologs, including *c3a.1, c3a.6, c3a.7-8, c3b.1,* and *c3b.2*, only *c3a.1* was significantly upregulated in neutrophils in response to wounding. Chromosomal duplication events in ancestors of teleost fish have resulted in striking isoform diversity in many complement components, including C3, and may represent an evolutionary strategy to enhance to efficacy of fish innate immunity in the face of a relatively under-developed adaptive immune system^47^. The gene products of the various C3 homologs differ structurally and in terms of their binding activity to various natural targets, including bacteria and fungi^20^. Further, cell-intrinsic complement has recently been demonstrated in multiple mammalian immune and non-immune cell types—most notably in T cells^12^, but also in macrophages^12^, epithelial cells^48^, and pancreatic β cells^49^. The non-canonical roles proposed for intracellular complement are diverse but include promotion of T cell effector activity upon migration to sites of inflammation, regulation of autophagy and cell survival, and mitochondrial antiviral signaling^12,14,48,49^. It is therefore plausible that neutrophil-specific transcription of *c3a.1* plays an autocrine role in neutrophil function, independent of other C3 isoforms or complement components. However, a specific role for C3 signaling, autocrine or otherwise, in neutrophil directed migration has not yet been fully established and requires further investigation.

Finally, we identified impaired neutrophil chemotaxis only in the very early post-wounding period (30 min post wound) in *c3a.1*^*−/−*^ larvae. Given the size of this gene and considering the known rates of eukaryotic transcription and translation^50,51^, rapid wound-induced transcriptional upregulation of *c3a.1* in neutrophils, as we detected using TRAP-RNAseq, may account for this early phenotype. This is in line with an earlier report showing that human neutrophils in culture upregulate transcription of C3 within 2 h of stimulation with either TNF-α or IFN-γ, although this does not result in secretion of C3 protein from the cell^52^. However, since *c3a.1*^*−/−*^ larvae lack c3a.1 expression both prior to wounding and in the post-wounding period, and since most complement proteins are produced at a basal level and activated by proteolysis upon immunologic insult, further investigation is needed to confirm a requirement for de novo* c3a.1* transcription in optimizing neutrophil chemotaxis to wounds. Nevertheless, our TRAP-RNAseq screening data serves as a useful starting point to identify promising genes for further study.

In summary, our data demonstrate the utility of large-scale TRAP screening to elucidate the cell type-specific gene expression changes that influence inflammation and wound healing. We identify the complement pathway as a whole, and *c3a.1* in particular, as significantly upregulated in neutrophils in response to wounding. Our data further support a zebrafish model with conserved C3 activity. We demonstrate a role for C3 in priming neutrophils for efficient migration to infection and wounds; however, the role of autocrine neutrophil C3 signaling warrants further investigation. On the basis of these observations, we conclude that C3 plays an underappreciated role in mediating neutrophil migration. By exploiting the genetic resources of the larval zebrafish model, we and others are well positioned to further investigate the role of neutrophil-derived C3 in optimizing neutrophil recruitment to wounding and to identify cell type-specific transcriptional responses that will add to our understanding of the signaling networks underlying leukocyte migration and wound healing.

## Methods

### Zebrafish lines, maintenance, and genotyping

All adult and larval zebrafish handling was carried out in full compliance of National Institute of Health guidelines and approved by the University of Wisconsin‐Madison Institutional Animal Care and Use Committee, as described previously^53^. Previously published zebrafish lines were used (Table 1). Larvae were anesthetized using 0.2 mg/mL tricaine before any experimentation. Zebrafish containing the mutant *c3a.1* allele sa31241 were isolated through the Sanger Zebrafish Mutation Project, Wellcome Sanger Institute, and obtained from the Zebrafish International Resource Center (ZIRC). This allele will be referred to as *c3a.1*^*−/−*^ herein. The sa31241 point mutation was detected by restriction fragment length polymorphism (RFLP) analysis. DNA was isolated in 50 mM NaOH, the mutated region amplified with GoTaq (Promega) (Table 2), and a restriction enzyme (DraI, NEB) directly targeting the mutant copy, but not the wild-type copy, was directly added with buffer. Digests were incubated overnight and run on a 2% agarose gel to evaluate the presence of mutant and/or wild-type bands.

**Table 1:** Published zebrafish lines used in this study.

Line	References
lyz:L10a-eGFP	^21^
mpeg1:L10a-eGFP	^21^
krt4:L10a-eGFP	^21^
mpeg1:eGFP	^18^
mpx:mcherry-2A-rac2	^29^
mpx:mcherry-2A-rac2^D57N^	^29^
mpx:dendra2	^35^
mpx:mcherry	^36^

**Table 2:** Primer sequences used in this study.

Primer	Sequence (5′ to 3′)	Ref. (if prev.published)
sa31241_F	TCACTCACGCTCTGTCTCTC	
sa31241_R	GGAAACATAGCTACTGACTGGA	
C3.1 qPCR F	TCCAGACAAGCGAAAGGTG	^23^
C3.1 qPCR R	CCATCAGTGTACACAGCATCATAC	^23^
C3.2/3 qPCR F	CGGTACACAAACACCCCTCT	^23^
C3.2/3 qPCR R	GTCTTCCTCATCGTTCTCTTGTT	^23^
C3.4 qPCR F	CAACTCAGAAGCGTCCATGA	^23^
C3.4 qPCR R	ATTGATCAGCCCTTGCAACT	^23^
C3.5 qPCR F	GTTGCACGCACAGACAAGTT	^23^
C3.5 qPCR R	CAGGCTCTTTCTCCATCTGC	^23^
C3.6 qPCR F	CAGACCACATCACTGCCAAC	^23^
C3.6 qPCR R	TTGTGCATCCGAAGTTGAAG	^23^
C3.7/8 qPCR F	CTCCATTTCGATGGCTGAAT	^23^
C3.7/8 qPCR R	ACATCACTCCGACCAGGAAC	^23^
C3b.1 qPCR F	TGAGATGGAGATTGTGCAGGT	
C3b.1 qPCR R	CTGCAGCTTGCATGAGAGAG	
C3b.2 qPCR F	CTGATCAGCATCAGCCAGAG	
C3b.2 qPCR R	CGCATGAGAGAGGAACATCC	
C5 qPCR F	CGGTTCAATCAGTGCTCAAA	
C5 qPCR R	TACTGCTTGCCAATCTCGAA	
EF1α qPCR F	TGCCTTCGTCCCAATTTCAG	Oehlers, Flores, et al. (2010)
EF1α qPCR R	TACCCTCCTTGCGCTCAATC	Oehlers, Flores, et al. (2010)

### Purification of mRNA from TRAP zebrafish larvae and RNA sequencing

3 dpf *Tg (lyz:EGFP-L10a), Tg(mpeg1:EGFP-L10a),* or *Tg(krt4:EGFP-L10a)*^9^ zebrafish larvae were anesthetized using 0.2 mg/mL tricaine and subjected to multiple tail fin wounds using a 33 gauge needle (Fig. 1A). A previously-published protocol for TRAP mRNA purification from zebrafish^9^ was performed, with slight modifications. Briefly, a QIAshredder (Quiagen) was used to homogenize the larvae prior to immunoprecipitation. mRNA was isolated from immunoprecipitated polysomes from 50 pooled wounded fish or unwounded controls using TRIzol reagent (Invitrogen). 4 paired replicates were collected. RNA quality and concentration was assessed on Agilent RNA PicoChip and samples with a concentration < 200 pg/µl were concentrated in a SpeedVac. As RNA concentrations were low, library prep was done with the NuGEN Ovation Single Cell RNA-Seq System with 20 cycles of amplification. Libraries were then checked by QuBit and an AATI Fragment Analyzer for concentration and fragment size. Adaptors with barcodes were used and samples were sequenced on an Illumina HiSeq with an average of 8 samples per lane. Sequencing generated 27.5 million single end reads per sample on average. Gene-level read counts were estimated using RSEM v1.2.20^54^ and Bowtie v1.1.1^55^ with the Ensembl v83 annotation of the GRCz10 assembly of the zebrafish genome. One *Tg (lyz:EGFP-L10a)* wounded sample was removed from the analysis during quality control assessments because it clustered most closely with other samples sequenced at the same time that had been generated via a different protocol.

Differentially expressed genes identified by RNA-seq were called using the DESeq2 R package^56^. The design formula for the generalized linear model used with DESeq2 was “ ~ replicate + condition” where “condition” was the combination of cell type and treatment for each sample. Statistical testing for differential expression within each cell type was performed using the Wald test implemented in the DESeq2 package. Translating RNAs with at least a twofold change in their relative abundance with a Benjamini–Hochberg corrected *P* value (FDR) ≤ 0.05 were considered statistically significant.

Human homologs of zebrafish genes were extracted from Ensembl using the BioMart tool. Gene Set Enrichment Analysis^22,57^ was performed by comparing gene expression data mapped to these human homologs to Hallmark gene sets (v6.2) from the Molecular Signatures Database (Broad Institute)^58^. The gsea3 java release was run using all default settings. Heatmaps were generated with Multiple Experiment Viewer (MeV) and Venn diagrams were generated and overlaps determined by BioVenn^59^.

TRAP-RNAseq results were verified by RT-qPCR quantification of *c3a.1* expression by neutrophils and macrophages in wounded and unwounded larvae. 3 dpf *Tg (lyz:EGFP-L10a)* or *Tg(mpeg1:EGFP-L10a* zebrafish larvae were anesthetized using 0.2 mg/mL tricaine and subjected to multiple tail fin wounds using a 33 gauge needle. TRAP-RNA purification was performed as above. RT-qPCR and fold change calculations were then performed as below. Primer sequences used in this study can be found in Table 2.

### RT-qPCR

RNA was extracted from approximately 50 pooled, 3 dpf *c3a.1*^+*/*+^ or *c3a.1*^*−/−*^ larvae using TRIzol reagent (Invitrogen). cDNA was then synthesized using SuperScript III RT and oligo-dT (Invitrogen). Using this cDNA as a template, quantitative PCR (qPCR) with FastStart Essential DNA Green Master (Roche) and a LightCycler96 (Roche) was performed. Fold changes in gene expression over control conditions, normalized to *ef1a*, were calculated from Cq values^60^. Primers used to amplify *c3a* orthologues^23^*,* and *ef1a*^61^ have been described previously. Due to high identity percentage, *c3a.2-3* and *c3a.7-8* were amplified and analyzed together, as previously described^23^. Primer sequences used in this study can be found in Table 2.

### *Pseudomonas* infections

3 dpf *c3a.1*^+*/*+^ or ^*−/−*^ larvae on a WT AB or *Tg(mpx:mcherry-2A-rac2*^*D57N*^) background^29^ were infected with *P. aeruginosa* PAK (pMF230, expresses GFP) as previously described^62,63^. PAK (pMF230) was a gift of Dara Frank (Medical College of Wisconsin). A single colony was inoculated overnight in LB. In the morning, the culture was diluted 1:5 and grown for an additional 1.25 h. The OD was measured (600 nm). The final inoculum was prepared by pelleting the bacterial suspension by centrifugation and resuspending the bacteria to achieve the desired bacterial density in 1X PBS containing 10% glycerol and 2% PVP-40 (to prevent needle clogging). Phenol red dye was added at a final concentration of 0.5% to visualize success of the injection. To monitor CFUs, the injection product was plated on LB and incubated overnight. Injected CFUs are noted in the figure legends. For survival experiments, infected larvae were placed into individual wells of a 96 well plate and survival was monitored daily for 5 dpi. For neutrophil recruitment experiments, larvae were fixed at 1 hpi or 6 hpi in 4% paraformaldehyde in 1X PBS overnight at 4 °C. Sudan Black B staining was performed as described previously^64^, and injection success was further confirmed by visualization of GFP-positive bacteria in the otic vesicle on a spinning disk confocal microscope (CSU-X, Yokogawa) as described below, without mounting in agarose. Imaging of the otic vesicle region for neutrophil enumeration was performed using a zoomscope (EMS3/SyCoP3; Zeiss; Plan-NeoFluar Z objective). Image analysis was performed using Zen 2012 (blue edition, Carl Zeiss).

### Tail transection

*C3a.1*^+*/−*^ adults were in-crossed. 3 dpf larvae were wounded by tail transection using a no. 10 Feather surgical blade. To visualize neutrophils in the wound microenvironment, the larvae were fixed at 2 hpw or 8 hpw in 4% paraformaldehyde in 1X PBS overnight at 4 °C. Sudan Black B staining was performed as described previously^64^. Fixed larvae were imaged using a zoomscope (EMS3/SyCoP3; Zeiss; Plan-NeoFluar Z objective) and then genotyped as above. For macrophage quantification, *c3a.1*^+*/−*^ adults carrying a *mpeg1:GFP* transgene^18^ were in-crossed. At 3 dpf, larvae were pre-screened for fluorescence on a zoomscope. Tail wounding was then performed as described above and the larvae were fixed in 1.5% formaldehyde overnight at 4 °C. Fixed larvae were imaged using a zoomscope and genotyped as above. All image analysis was performed using Zen 2012 (blue edition, Carl Zeiss), blinded to genotype.

### Photoconversion

Adult *c3a.1*^+*/-*^ zebrafish carrying an *mpx:Dendra2* transgene^35^ were in-crossed and embryos collected and incubated to 3 dpf. Larvae were prescreened for fluorescence using a zoomscope (EMS3/SyCoP3; Zeiss; Plan-NeoFluar Z objective) and mounted in ZWEDGI devices as previously described^65^. An imaging sequence was performed for each larva comprising an initial series of 2 overlapping Z-stacks of the region of the caudal hematopoietic tissue (CHT) and photoconversion of the neutrophils within the CHT. This was followed by a second series of 2 overlapping Z-stacks to confirm that photoconversion occurred. Photoconversion was performed using a laser scanning confocal microscope (FluoView FV1000; Olympus) with numerical aperature (NA) 0.75/20X objective. The following stimulation settings were used: 40% 405 nm laser transmissivity, 10 µs/pixel dwell time, and 45 s total stimulation time. Larvae were removed from the ZWEDGI devices following photoconversion and subjected to wounding by tail transection as above. Larvae were subsequently imaged live at 3 hpw using a spinning disk confocal microscope as described below and then genotyped as above. Image analysis was performed using Zen 2012 (blue edition, Carl Zeiss), blinded to genotype.

### Live imaging and image quantification

3 dpf *c3a.1*^+*/*+^ or ^*−/−*^ larvae carrying a *mpx:mcherry* transgene^36^ were pre-screened for fluorescence on a zoomscope (EMS3/SyCoP3; Zeiss; Plan-NeoFluar Z objective). For imaging over 1–3 h, larvae were mounted in ZWEDGI devices as previously described^65^ and retained in place using 2% low melting point agarose applied to the head. Images were acquired every 3 min using a spinning disk confocal microscope (CSU-X, Yokogawa, NA 0.3/10X EC Plan-NeoFluar objective) with a confocal scanhead on a Zeiss Observer Z.1 inverted microscope equipped with a Photometrics Evolve EMCCD camera. Each image comprised a 50 µm z-stack, with 11 slices taken at 5 µm intervals. Images were analyzed and maximum intensity projections were made using Zen 2012 (blue edition) software (Carl Zeiss). To track cell motility, time series were analyzed in Imaris (Bitplane) and neutrophil mean track speed, track displacement, and track straightness, as well as instantaneous velocity for each neutrophil at each point in the time series, were calculated using the “spots” tool as previously described^66^. To count total neutrophils and quantify neutrophil distribution in photoconversion experiments, 12 overlapping images were acquired to capture the full length and width of each larva and image analysis and neutrophil counts were performed using the “events” tool in Zen 2012 (Blue edition, Carl Zeiss).

### Statistical analyses

For RT-qPCR gene expression analyses, 3 independent biologic replicate experiments were performed. Each experiment comprised 3 technical replicates. Statistical significance was determined by comparing the calculated ΔCq of the experimental conditions using the non-parametric Wilcoxon two-group test^67^. Fold change in ΔΔCq calculated and plotted in terms of mean and standard error.

For neutrophil quantification and migration analyses, 3–4 independent replicate experiments were performed. Replicate numbers are noted in the figure legends. Experimental conditions were compared using analysis of variance. The results were summarized and plotted in terms of least squares adjusted means and standard errors.

For survival curves, 3 independent experiments were performed. Results were pooled and analyzed by Cox proportional hazard regression analysis, with experimental conditions included as group variables. Statistical analyses were performed using R version 3.4.4 and graphical representations were made using GraphPad Prism version 7. Significance was defined as *P* < 0.05. The resulting *P* values are included in the figure legends for each experiment.

For quantification of neutrophil instantaneous speed over time, a linear mixed effect regression model was used. Genotype and time were treated as fixed effects, with experimental replicate, fish, and neutrophil (within fish) treated as random effects. Statistical analyses were performed in R version 3.5.1, using the associated lme4 package. Reported *P* values are 2-sided and level of statistical significance preset to 0.05, with no adjustment for multiplicity.

## Supplementary information

Supplementary Legends

Supplementary Movie

Supplementary Table

Supplementary Figure1

Supplementary Figure2

Supplementary Figure3

## Data Availability

The datasets generated or analyzed by this study are included this article and its supplementary information files or are available from the corresponding author upon reasonable request.
