# Supplementary material for: Cell type specific gene expression profiling reveals a role for complement component C3 in neutrophil responses to tissue damage
Source: Sci Rep. 2020 Sep 24;10:15716. doi: 10.1038/s41598-020-72750-9 (PMC7518243; doi:10.1038/s41598-020-72750-9)
Supplement: Supplementary file 1 — Supplementary Legends [file 41598_2020_72750_MOESM1_ESM.docx]

**Supplemental Material**

**Supplemental Table 1:** Differentially expressed genes shown in Figure 1 C.

**S1:** Global depletion of *c3a.1* does not alter macrophage recruitment to tail wounds. Quantification of macrophage numbers at the wound following tail transection of *c3a.1^+/+^* (n = 15, 4hpw; 10, 24hpw) or *c3a.1^-/-^* (n = 19, 4hpw; 17, 24hpw) *Tg(mpeg1:GFP)* larvae, expressed as mean +/-SEM. Each dot represents one larva; colors represent the results of 3 independent experiments.

**S2.** (A) CHT neutrophils of *Tg(mpx:dendra2)* *c3a.1^+/+^* or *c3a.1^-/-^* were photoconverted and the larvae subjected to tail transection. (B) Quantification of photoconverted neutrophils retained in the CHT at 3hpw. (C) Quantification of photoconverted neutrophils outside the CHT at 3hpw. *C3a.1^+/+^* (n=16); *c3a.1^-/-^* (n=22). All data are expressed as mean +/- SEM, with each dot representing one larva and colors representing 4 independent experiments.

**Movie 1.** Representative time-lapse movies of recruitment of mcherry-labeled neutrophils to tail-transected caudal fins of *c3a.1^+/+^* (left) or *c3a.1^-/-^* (right) zebrafish larvae, 0-60 minutes post-wound. Tracks of forward-migrating neutrophils are labeled in color.

**S3.** (A) Quantification of mean linear displacement distance traveled by forward-migrating neutrophils in *c3a.1^+/+^* (n = 8) and *c3a.1^-/-^* (n = 8) during early neutrophil recruitment. (B) Mean track straightness of forward-migrating neutrophils during early neutrophil recruitment does not differ between *c3a.1^+/+^* (n = 8) and *c3a.1^-/-^* (n = 8) larvae. (C) Quantification of mean track speed of randomly migrating neutrophils in the heads of unwounded *c3a.1^+/+^* (n = 9) or *c3a.1^-/-^* (n = 8) larvae. All data are expressed as mean +/- SEM. Each dot represents the mean of value for all neutrophils for one larva. Colors represent results of 4 independent experiments. *p<0.05.
