# Supplementary figures and images for: Cell type specific gene expression profiling reveals a role for complement component C3 in neutrophil responses to tissue damage
Source: Sci Rep. 2020 Sep 24;10:15716. doi: 10.1038/s41598-020-72750-9 (PMC7518243; doi:10.1038/s41598-020-72750-9)

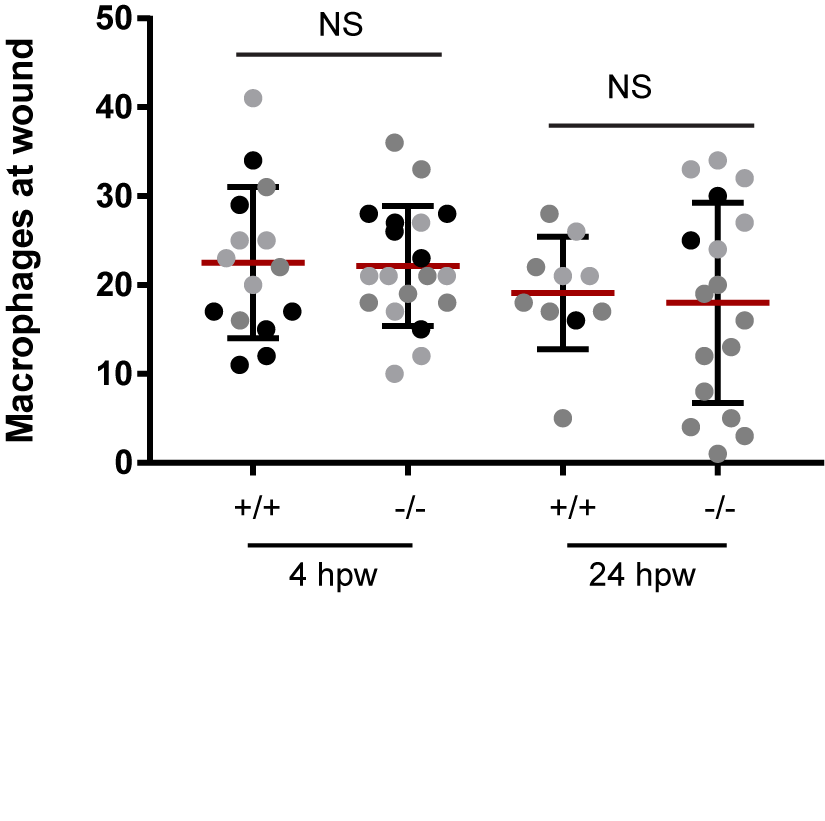

Supplement: Supplementary file 4 — Supplementary Figure1 [file 41598_2020_72750_MOESM4_ESM.tif]

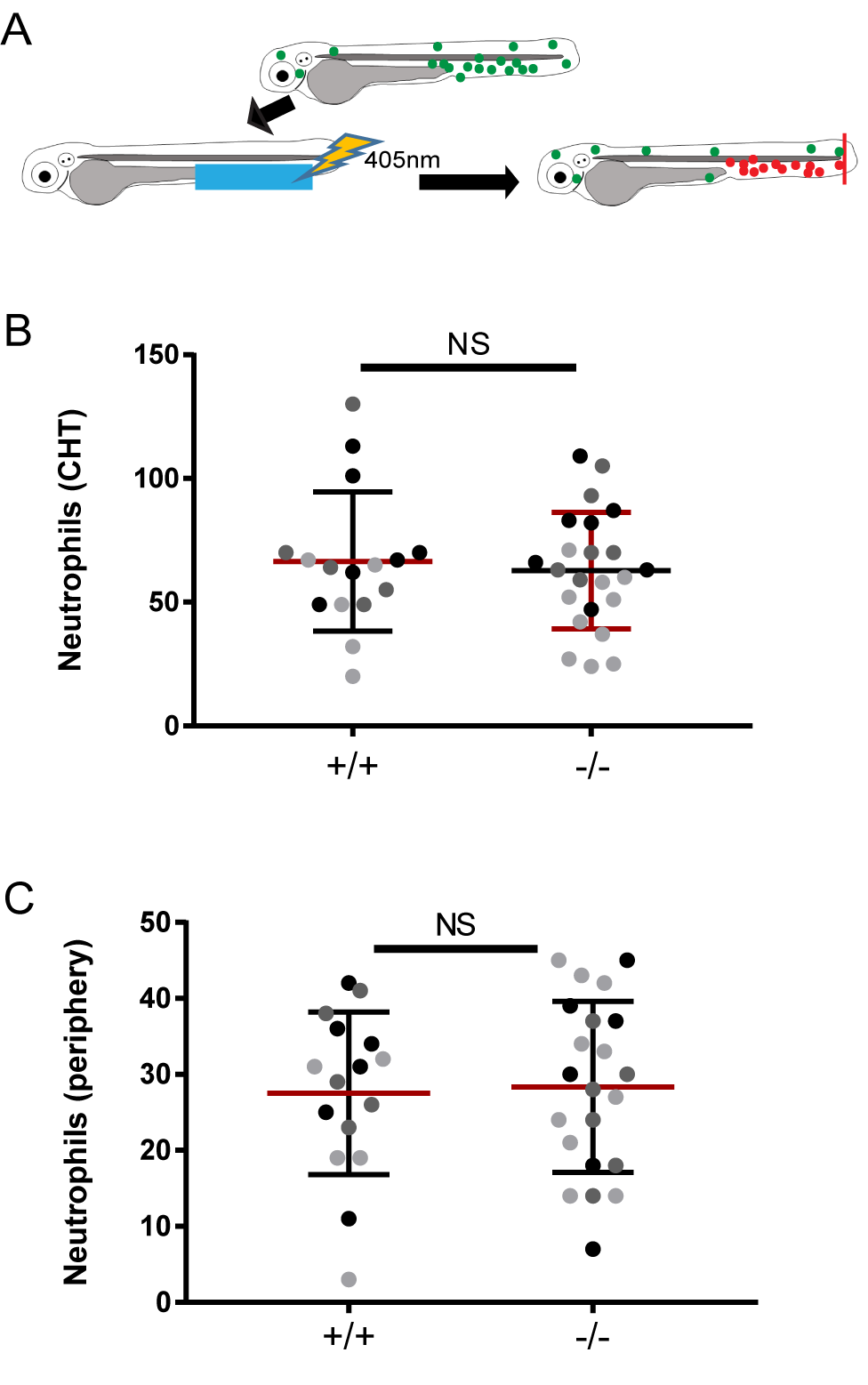

Supplement: Supplementary file 5 — Supplementary Figure2 [file 41598_2020_72750_MOESM5_ESM.tif]

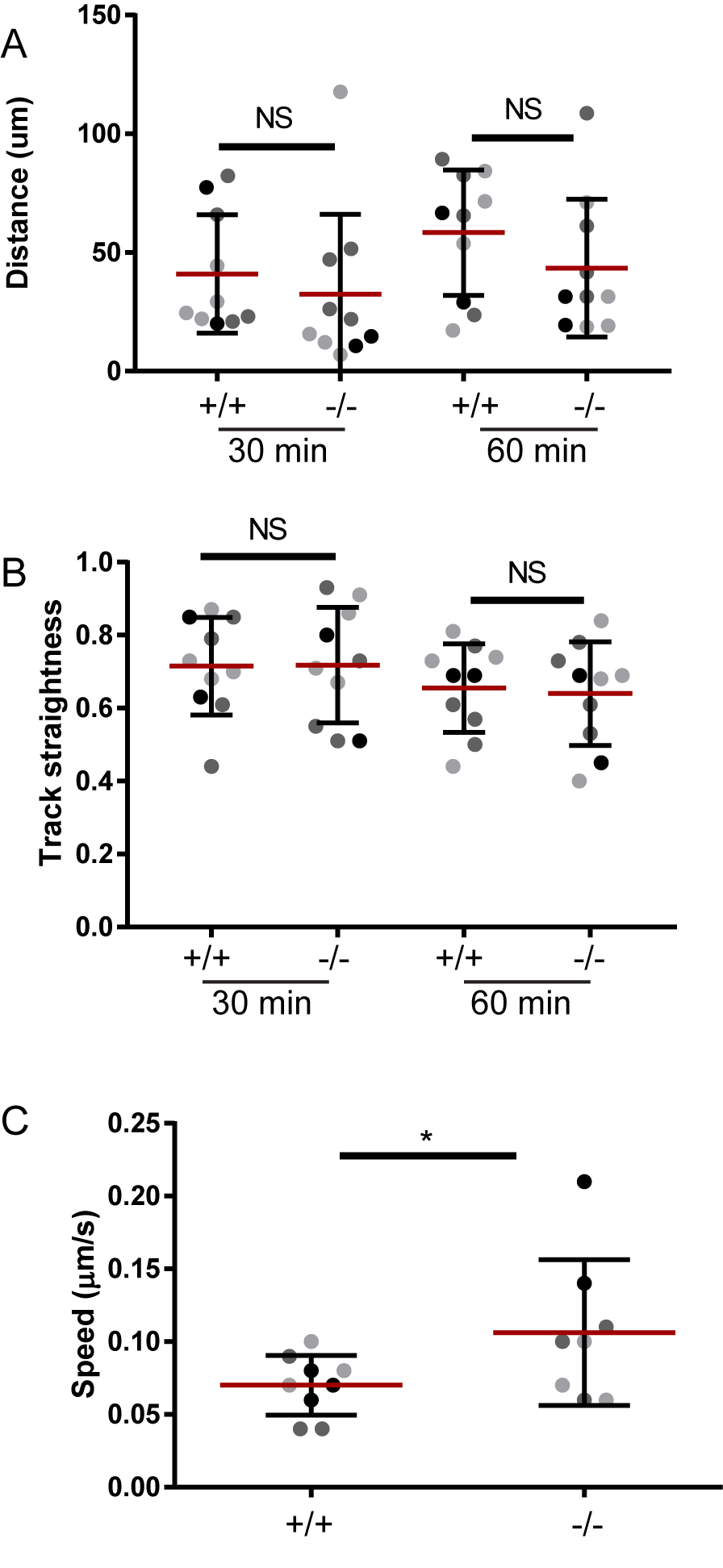

Supplement: Supplementary file 6 — Supplementary Figure3 [file 41598_2020_72750_MOESM6_ESM.tif]
